# LGMD2I in a North American population

**DOI:** 10.1186/1471-2474-8-115

**Published:** 2007-11-24

**Authors:** Peter B Kang, Chris A Feener, Elicia Estrella, Marielle Thorne, Alexander J White, Basil T Darras, Anthony A Amato, Louis M Kunkel

**Affiliations:** 1 Program in Genomics, Children's Hospital Boston and Harvard Medical School, Boston, USA; 2 Department of Neurology, Children's Hospital Boston and Harvard Medical School, Boston, USA; 3 Howard Hughes Medical Institute, Children's Hospital Boston, Boston, USA; 4 Department of Neurology, Brigham and Women's Hospital and Harvard Medical School, Boston, USA

## Abstract

**Background:**

There is a marked variation in clinical phenotypes that have been associated with mutations in *FKRP*, ranging from severe congenital muscular dystrophies to limb-girdle muscular dystrophy type 2I (LGMD2I).

**Methods:**

We screened the *FKRP *gene in two cohorts totaling 87 patients with the LGMD phenotype.

**Results:**

The c.826C>A, p.L276I mutation was present in six patients and a compound heterozygote mutation in a seventh patient. Six patients had a mild LGMD2I phenotype, which resembles that of Becker muscular dystrophy. The other patient had onset before the age of 3 years, and thus may follow a more severe course.

**Conclusion:**

These findings suggest that LGMD2I may be common in certain North American populations. This diagnosis should be considered early in the evaluation of LGMD.

## Background

Fukutin-related protein (FKRP) is encoded by the gene *FKRP *and participates in the glycosylation of α-dystroglycan in the muscle fiber[1]. There is evidence to suggest that the protein localizes to both the Golgi apparatus[1] and the endoplasmic reticulum[2], with some debate over the possibility that mislocalization of FKRP plays a role in the pathogenesis of disease [3-5]. Mutations in *FKRP *can cause a range of phenotypes, including MDC1C (a severe congenital muscular dystrophy) [6], Walker-Warburg syndrome[7], muscle-eye-brain disease[7], a severe form of limb-girdle muscular dystrophy type 2I (LGMD2I), and a mild form of LMGD2I[8]. One particular mutation, c.826C>A, p.L276I, is by far the most common mutation causing LGMD2I, present either in homozygote form or as part of a compound heterozygote genotype [8-12]. Since the original descriptions of these associations, it has become evident that LGMD2I is one of the more common LGMDs in the United Kingdom [13], Denmark[10], and Brazil[12]. A recent series also suggests the same for the United States[14]. We have similarly found that LGMD2I may be one of the more common LGMDs in two North American cohorts.

## Methods

In the first cohort, 63 patients were ascertained either prospectively at the Children's Hospital Boston Neuromuscular Program or by referral from other neurologists in North America for the possible diagnosis of LGMD. Clinical data collected included age of onset, pattern of muscle involvement, family history, creatine kinase (CK) level in serum, and cardiac involvement. All subjects had progressive muscle weakness and myopathic findings on muscle biopsy. We enrolled patients without a known molecular etiology into the first cohort under an institutionally approved protocol. Informed consent was obtained from all patients in this cohort. In the second cohort, one of the authors (AAA) screened 24 patients from his clinic with the clinical diagnosis of LGMD for mutations in *FKRP *and other genes known to cause LGMD, using clinically available genetic testing for mutations in *CAPN3*, *DYSF*, and *CAV3*. Facioscapulohumeral muscular dystrophy and Duchenne/Becker muscular dystrophies were excluded by genetic testing. Dysferlinopathies were also excluded in the second cohort by Western blot. In both cohorts, patients thought to have Duchenne or Becker muscular dystrophy without known mutations were included. Many of the enrolled patients had muscle biopsies performed at other centers, and thus the immunohistochemistry stains obtained vary from patient to patient. The methods described below apply to the first cohort.

For exon 4 amplification, the primers used were agctgctggacttgaccttc (forward) and tccaagtagatgcccaggtc (reverse). The Fast Start Taq DNA Polymerase Kit (Roche Diagnostics, Pleasanton, CA) was used to set up the PCR reactions, which were run at 95°C for 4 minutes, followed by 35 cycles of 95°C for 30 seconds, 60°C for 30 seconds, and 72°C for 2 minutes, followed by 72°C for 7 minutes.

For the exon 4 sequencing reaction, the primers used were ccgagtttgtggccctagta (forward) and ccagccttctctcatgctct (reverse). The sequences were visualized on the 3730 DNA Analyzer (Applied Biosystems, Foster City, CA) and interpreted using the Sequencher 4.6 software program (Gene Codes Corporation, Ann Arbor, MI). Positive results were confirmed on separate aliquots of DNA that were processed and analyzed in a CLIA-approved DNA diagnostic facility.

Fifty-six of the patients in cohort 1 were also screened for mutations in *CAV3 *using direct sequencing methods (primers kindly contributed by RR Bennett).

## Results

Among the 63 LGMD patients in the first cohort, 40 were male and 23 female. The age of onset ranged widely from birth to 70 years. The ethnic backgrounds included 38 Caucasians, 7 Hispanic-Americans, 5 African-Americans, 4 Asian-Americans, and 9 unknown. Four patients in the first cohort, all from the United States, were diagnosed with LGMD2I based on mutations in *FKRP*. Among the 24 LGMD patients in the second cohort, 3 were diagnosed with LGMD2I, 8 with Becker muscular dystrophy, 2 with LGMD2A, 5 with dysferlinopathy (3 with LGMD2B, 2 with Miyoshi myopathy), 1 with FSHD (facial sparing), and 5 are undiagnosed to date.

Demographic and clinical information on the seven patients diagnosed with LGMD2I are listed in Table 1. Patient 1 had onset of symptoms as a toddler, while the others developed symptoms later in childhood or in early adulthood. Age of onset was defined as the age when patients recalled first having motor difficulties, not the age of presentation. None had significant motor delays. Each patient was sporadic, consistent with a recessive pattern of inheritance. The pattern of weakness was predominantly proximal, with the lower extremities more severely affected than the upper extremities. Facial strength was generally preserved, while mild scapular winging was present in some patients. Evidence of mild cardiac or respiratory dysfunction was present in some patients. All of the patients were ambulatory at the last known follow-up. Muscle biopsy was performed in patients 1 through 6. Histochemical stains demonstrated variation in fiber size, degenerating and regenerating fibers, increased endomysial or perimysial connective tissue, and fiber splitting. Immunohistochemical findings are listed in Table 1.

**Table 1:** Clinical features of patients with FKRP mutations

	Patient 1	Patient 2	Patient 3	Patient 4	Patient 5	Patient 6	Patient 7
Gender	Male	Male	Female	Male	Female	Female	Male
Ethnicity	-	Causasian	Caucasian	Caucasian	Caucasian	Caucasian	Asian
Onset	34 months	1^st ^grade	18–19 years	Childhood	23 years	Childhood	25 years
Chief complaint	Difficulty rising	Difficulty running	Exertional myalgias	Difficulty running	Difficulty with stairs	Difficulty running	Difficulty with stairs
Calf pseudohypertrophy	Present	-	None	Present	None	Present	Medial calf atrophy
Heel cords	Contracted	-	Normal	Unknown	Contracted	Normal	-
Facial strength	Normal	Mild weakness	Normal	Normal	Normal	Normal	Normal
Scapular winging	-	-	-	-	Subtle	Subtle	Subtle
Proximal upper extremity strength	-	4+	4-	5	4-	4	4+
Distal upper extremity strength	-	5	5	5	4+ to 5	4+	5
Proximal lower extremity strength	Weak	2+ to 3+	3- to 4-	2 to 3	2	3- to 3	3- to 4-
Distal lower extremity strength	-	5	5	5	4	4	5- to 5
Reflexes	2+	Trace	2+	1+ to 2+	1+ to 2+	2+	2+
FVC (% predicted)	-	-	-	-	2.72L (83%)	2.87L (75%)	3.03L (56%)
Echocardiogram (ejection fraction)	Normal	-	Normal (55%)	Angiogram normal	Normal (>55%)	~45%	(>55%)
Creatine kinase (U/L)	5,438	5,000+	1,945	3–4 times normal	1,136	5,031	6,560 to 10,642
Dystrophin duplication/deletion	None	-	None	None	-	-	-
Age at biopsy	3 1/2 years	17 years	30 years	51 years	27 years	25 years	-
Muscle sampled	Left quadriceps	Left biceps	Deltoid	Left quadriceps	Biceps	Deltoid	-
Dystrophin staining	Normal	Normal	Normal	Normal	Normal	Normal	-
Merosin staining	Normal	-	-	-	-	Decreased	-
Sarcoglycans (α,β,γ,δ)	Normal	Normal	Normal	-	Normal	Normal	-
β-dystroglycan	Normal	-	Normal	-	Normal	Normal	-
Spectrin	-	Normal	Normal	-	Normal	Normal	-
FKRP mutation	c.826C>A, p.L276I (homozygous)	c.826C>A, p.L276I (homozygous)	c.826C>A, p.L276I (homozygous)	c.826C>A, p.L276I (homozygous)	c.826C>A, p.L276I (homozygous)	c.826C>A, p.L276I (homozygous)	del 1006–1174 (169 basepair frameshift, heterozygous), c.328C>T, p.R110W (heterozygous)

-, not done or not assessed; FVC, forced vital capacity; extremity strength graded on Medical Research Council (MRC) scale

The first six patients described in Table 1 have homozygous c.826C>A, p.L276I mutations in *FKRP*. The seventh patient is a compound heterozygote, with a 169 base pair deletion in one allele and a c.328C>T, p.R110W mutation in the other allele. The entire coding region of FKRP was screened in the remaining 59 patients from the first cohort who did not have the common mutation. No other mutations were identified. No mutations in *CAV3 *were identified in either cohort.

## Discussion

It is becoming increasingly clear that LGMD2I, caused by mutations in *FKRP*, is one of the more common LGMDs, possibly rivaling LGMD2A (calpainopathy) in prevalence[10]. In particular, the homozygous c.826C>A, p.L276I mutation is the most common *FKRP *mutation in several series of Caucasian populations [8-10,12-15]. There is only one study to date that suggests otherwise, listing compound heterozygotes involving the c.826C>A mutation as being most abundant[11]. The homozygous form of the common mutation causes the milder phenotype of LGMD2I with onset in childhood or early adulthood and a slow progression. Ambulation appears to be preserved until at least middle age, suggesting that some affected individuals may not seek medical attention and may not be diagnosed due to the mild nature of their symptoms. The more severe phenotypes (severe LGMD2I, MDC1C, Walker-Warburg syndrome, and muscle-eye-brain disease) are generally caused by other mutations. There have been recent reports of patients with LGMD2I having previously been diagnosed incorrectly with a dystrophinopathy, thus it is important to confirm a molecular diagnosis for any of the muscular dystrophies[9].

A clinical comparison of the patients in our series to those in others is best made by focusing on the patients who have the common homozygous c.826C>A mutation[8-10,12,13]. The clinical courses of these patients to date correlate well across the series, especially with regard to the preservation of ambulation in most individuals into middle adulthood. The age of onset in several of our patients is at the earlier end of the range observed previously. In our series, cardiac and pulmonary complications are present in a minority of patients, consistent with another series[15]. However, some of our subjects have not yet been screened for these conditions, and other series suggest a high prevalence of both complications[16,17].

## Conclusion

LGMD2I may be one of the more common forms of LGMD in some North American populations. The most common mutation generally cause a mild phenotype, but these patients have clearly elevated creatine kinase levels, making it easy to screen for this disorder in patients with mild gait difficulties. As further cohorts of LGMD patients are analyzed, the incidence of each subtype will become more clearly defined.

## List of abbreviations used

FKRP, fukutin-related protein

LGMD, limb-girdle muscular dystrophy

MDC1C, congenital muscular dystrophy type 1C

## Competing interests

The author(s) declare that they have no competing interests.

## Authors' contributions

All authors have read and approved the final manuscript.

PBK helped select subjects to be studied, analyzed data, and drafted and edited the manuscript.

CAF performed PCR reactions, prepared sequencing reactions, analyzed data, and helped edit the manuscript.

EE managed patient databases, helped select subjects to be studied, and helped edit the manuscript.

MT managed patient databases, advised on technical issues, and helped analyze data.

AJW performed PCR reactions, prepared sequencing reactions, and analyzed data.

BTD contributed subject information and clinical background, and helped edit the manuscript.

AAA contributed subject information and clinical background, and helped edit the manuscript.

LMK supervised the project, providing advice and guidance on all aspects of the study, and helped edit the manuscript.

## Pre-publication history

The pre-publication history for this paper can be accessed here:


